# Low Residual CBF Variability in Alzheimer’s Disease after Correction for CO_2_ Effect

**DOI:** 10.3389/fnene.2012.00008

**Published:** 2012-07-05

**Authors:** Anders Bertil Rodell, Joel Aanerud, Hans Braendgaard, Albert Gjedde

**Affiliations:** ^1^Department of Nuclear Medicine and Positron Emission Tomography Centre, Aarhus University HospitalAarhus, Denmark; ^2^Department of Neurology, Aarhus University HospitalAarhus, Denmark; ^3^Department of Neuroscience and Pharmacology and Center of Healthy Aging, Faculty of Health and Medical Sciences, University of CopenhagenCopenhagen, Denmark

**Keywords:** Alzheimer’s disease, brain energy metabolism, cerebral blood flow, cerebral metabolic rate for oxygen, cerebral vasoreactivity

## Abstract

We tested the claim that inter-individual CBF variability in Alzheimer’s disease (AD) is substantially reduced after correction for arterial carbon dioxide tension (PaCO_2_). Specifically, we tested whether the variability of CBF in brain of patients with AD differed significantly from brain of age-matched healthy control subjects (HC). To eliminate the CO_2_-induced variability, we developed a novel and generally applicable approach to the correction of CBF for changes of PaCO_2_ and applied the method to positron emission tomographic (PET) measures of CBF in AD and HC groups of subjects. After correction for the differences of CO_2_ tension, the patients with AD lost the inter-individual CBF variability that continued to characterize the HC subjects. The difference (Δ*K*_1_) between the blood-brain clearances (*K*_1_) of water (the current measure of CBF) and oxygen (the current measure of oxygen clearance) was reduced globally in AD and particularly in the parietal, occipital, and temporal lobes. We then showed that oxygen gradients calculated for brain tissue were similar in AD and HC, indicating that the low residual variability of CBF in AD may be due to low functional demands for oxidative metabolism of brain tissue rather than impaired delivery of oxygen.

## Introduction

### CBF variability

Cerebral blood flow (CBF) measures vary greatly among healthy individuals at rest (Ito et al., 2004; Aanerud et al., 2012). Some of this inter-individual variability may arise from intra-individual temporal fluctuations in CBF regulation, such as arterial CO_2_ tension changes or varying functional demands within each subject. At subject level, intra-individual measures of CBF variability also provide the basis for current functional brain mapping techniques with [^15^O]water in PET, and also with BOLD signals in functional Magnetic Resonance Imaging (fMRI). The inter-individual CBF variation among healthy people and patients with neurodegenerative disorders such as Alzheimer’s disease can therefore arise from separate classes of variation: (i) Individual transient fluctuations varying with time constants of seconds, minutes, or hours, and (ii) Intrinsic variability among the subjects’ ability to regulate blood flow. Separating these classes of variation becomes crucial in determining pathological conditions affecting either (i) or (ii) and in determining their causal relationship.

In this work we (A) develop a new method for estimating the change in CBF in response to PaCO_2_ (which is a well described regulator of CBF), and (B) we show that once the CO_2_ variation is factored out, there remains very little variation in a group of patients suffering from AD compared to a group of healthy aged control subjects. To analyze possible links between the loss of variability in AD and brain metabolism, we also (C) analyze the relationship between CBF and oxygen consumption CMRO_2_.

### Factors influencing CBF variability

Some factors influencing the variability of CBF are known, including arterial blood pressure changes and variations of the arterial carbon dioxide tension (PaCO_2_), but the mechanisms of global and regional flow-metabolism coupling in response to changes of brain functional demands, embodied in part in the so-called neurovascular control unit, involving both microvessels and astrocytic endfeet, are unclear, despite many years of study (Berne et al., 1981; Kontos, 1981; Nicolakakis and Hamel, 2011; Peterson et al., 2011).

Changes in the cerebral microvascular endothelium may be implicated in the pathogenesis of neurodegenerative diseases and possibly even in the onset of AD, as regulatory deficiency can cause cerebral hypoperfusion which may precede or contribute to AD (Nagata et al., 2000; Farkas and Luiten, 2001; Grammas et al., 2011; van Beek et al., 2012). Thus, the evidence of amyloid-related pathogenesis of AD may bear directly on endothelial function (Deane et al., 2003; Grammas et al., 2011) and indirectly on the transient control of CBF, although the order of cause and effect remains uncertain (Chen et al., 2011). Indeed, the findings suggest that loss of vasomotor dynamics may contribute to the hypoperfusion, if the neurovascular mediators of functional flow variability are uncoupled from the response to CO_2_ (Iliff et al., 2003).

### Pressure autoregulation of CBF

In the following, we distinguish between purely vascular reactivity, which includes pressure autoregulation and CO_2_ response, on one hand, and the metabolic or functional reactivity, on the other (Nagata et al., 2000). Blood pressure autoregulation serves to minimize the effect of arterial blood pressure changes on cerebral blood flow. The mechanism is uncertain, but it differs fundamentally from the process that elicits changes of cerebral blood flow in response to changes of CO_2_. Thus, situations exist in which one of the two mechanisms is preserved and the other is impaired (Lauritzen, 1984; Panerai et al., 1999).

With transcranial laser Doppler technique in patients with mild AD, Claassen et al. found little impairment of cerebral autoregulation, but generally lower variability of CBF velocity (CBFV; Claassen et al., 2009; Claassen and Zhang, 2011). The low variability was reflected in the average baseline CBFV measure of 38 vs. 55 cm/s in AD vs. HC, although the authors later were unable to confirm the findings (van Beek et al., 2012). Using PET in AD, Zazulia et al. (2010) found that cerebral perfusion rates remained stable with a moderate 14% decline of blood pressure, although interpretations were complicated by the low blood pressure change and the use of nicardipine to modify the blood pressure (Claassen and Zhang, 2011).

### CO_2_ effect on CBF

The vasoactive response to carbon dioxide was established by Carl F. Schmidt as early as in 1928 and by the later reports of Kety and Schmidt on the effects of CO_2_ inhalation in pilots (Schmidt, 1928; Kety and Schmidt, 1946, 1948), followed by extensive confirmation until this date. Evidence suggests that CO_2_ reactivity is preserved in human AD (Jagust et al., 1997; Nagata et al., 2000), and likewise in transgenic mice that overexpress the amyloid precursor protein (APP; Niwa et al., 2002).

### Corrections for CO_2_ effect

The findings above suggest that the low, but relatively constant baseline perfusion in AD possibly is related to reduced activity of the neurovascular mediators of fluctuating functional demands. Thus, a fundamental loss of functional brain dynamics in AD can be revealed only when CBF variations related to PaCO_2_ change are eliminated. Here, we reveal the degree of reduction of the variability of CBF remaining after elimination of the CO_2_ effect. We use a novel approach to correction for PaCO_2_ changes, which corrects both individual and group measures of CBF, in relation to the average normocapnic PaCO_2_ and its corresponding CBF. The method as presented has broad applicability to CBF measurements, regardless of modality, including non-invasive measures of CO_2_ (for example with a finger monitor). We used this novel approach to determine how much of the CBF variability remained after the reactivity due to variations of PaCO_2_ was eliminated in a group of healthy individuals and a group of patients with AD.

### Delivery of oxygen after loss of variability

To understand how the loss of CBF variability related to functional demands in the AD patients, we tested whether the dependence of oxygen consumption on oxygen delivery to the brain (Δ*K*_1_) was affected in brain of the patients with AD.

## Materials and Methods

The methods used in this study consist of three major parts

1) Derivation of the method used for factoring out the CO_2_ variability, and how to use it for other studies.
2) Methods pertaining to the acquisition of the data from the AD and HC subjects.
3) How to combine the CBF and CMRO_2_ data in order to estimate the oxygen gradient in the tissue.

### Correction for CO_2_ effect

The method used for factoring out the CO_2_ variability in humans was developed by modifying the functional relationship of CBF derived from a primate study, so that it fits well with the human response to CO_2_ changes in the blood. The regression for estimating the human parameters was done using previous published studies. The developed method^1^ can be generally applied to any new dataset where normocapnic set-point of mean CBF and mean CO_2_ are known.

#### Estimating the CO_2_ effect in humans from historic data

The correction factor *f_C_* is the relative change that an average baseline CBF value measured at a standard PaCO_2_ level undergoes as a function of a varying PaCO_2_ in the blood [see equation (8) below].

Corresponding single subject values of PaCO_2_ and CBF in hypo-, normo-, and hypercapnia of humans were extracted from values reported by Kety and Schmidt (1946, 1948), and digitized from Figure 2 reported by Ramsay et al. (1993). Normalized fractional values were calculated relative to the mean normal values presented by Ramsay et al. (1993). For each subject, the relative fractional hypocapnic and hypercapnic values of PaCO_2_ were converted to units of mmHg relative to the mean normocapnic PaCO_2_ tension of 39.5 mmHg. The single subject fractional CBF response was calculated relative to the normocapnic CBF value, as listed in Table 1. The relative PaCO_2_ values and fractional CBF response were used to estimate the correction factor *f*_C_. To do so, we considered several possible model functions of the data and chose the exponential relation given by Reivich (1964). In order to model the data for use as a practical correction, we modified the parameters of the shape of the response curve of Reivich (1964) to reflect the relative human CBF measures as function of the PaCO_2_ variation relative to the mean normocapnic PaCO_2_. The relationship presented as Figure 3 of Reivich (1964) for rhesus monkeys is a logistic curve in which the lower limit of hypocapnic CBF is 20.9 mL/100 g/min and the upper limit of hypercapnic CBF is 113.7 mL/100 g/min. By normalizing to the average measured baseline CBF of 49.3 mL/100 g/min in the present study, the relative relationship $f_{C}^{\text{rhesus}}$ for rhesus monkeys became,

**Table 1:** **Carbon dioxide reactivity**.

	PaCO_2_ mmHg			Relative PaCO_2_ mmHgv			CBF mL/min 100 g			CBF fractional		
	H	C	E	*FH*/*C*	*FC*/*C*	*FE*/*C*	H	C	E	H/C	C/C	E/C
**Ramsay et al. (1993)**
Gray matter	24	38	55	24.9	39.5	57.2	45	73	115	0.62	1.00	1.58
	23	38	55	23.9	39.5	57.2	37	50	105	0.74	1.00	2.10
	24	41	53	23.1	39.5	51.1	32	47	108	0.68	1.00	2.30
	20	40	55	19.8	39.5	54.3	43	68	118	0.63	1.00	1.74
	20	42	57	18.8	39.5	53.6	30	51	75	0.59	1.00	1.47
	20	38	55	20.8	39.5	57.2	49	52	104	0.94	1.00	2.00
White matter	24	38	55	24.9	39.5	57.2	18	16	28	1.13	1.00	1.75
	23	38	55	23.9	39.5	57.2	12	20	35	0.60	1.00	1.75
	24	41	53	23.1	39.5	51.1	15	25	39	0.60	1.00	1.56
	20	40	55	19.8	39.5	54.3	10	21	39	0.48	1.00	1.86
	20	42	57	18.8	39.5	53.6	10	22	32	0.45	1.00	1.45
	20	38	55	20.8	39.5	57.2	15	20	32	0.75	1.00	1.60
Mean	21.8	39.5	55	21.8	F = 39.5	55.0						
**Kety and Schmidt (1946)**
Active- hyper- ventilation	31	52		23.5	39.5		44	59		0.75	1.00	
	28	55		20.1	39.5		51	87		0.59	1.00	
	29	43		26.6	39.5		40	70		0.57	1.00	
	32	41		30.8	39.5		48	56		0.86	1.00	
	24	44		21.5	39.5		40	69		0.58	1.00	
	25	43		23.0	39.5		52	79		0.66	1.00	
Passive- hyper- ventilation	22	38		22.9	39.5		40	59		0.68	1.00	
	23	42		21.6	39.5		44	62		0.71	1.00	
	30	46		25.8	39.5		47	72		0.65	1.00	
	27	45		23.7	39.5		40	81		0.49	1.00	
	22	45		19.3	39.5		36	55		0.65	1.00	
Mean	26.6	44.9										
**Kety and Schmidt (1948)**
5% CO_2_		42	50		39.5	47.0		48	65		1.00	1.35
		42	47		39.5	44.2		50	67		1.00	1.34
		41	48		39.5	46.2		46	75		1.00	1.63
		48	54		39.5	44.4		63	90		1.00	1.43
		41	46		39.5	44.3		56	80		1.00	1.43
		42	53		39.5	49.8		63	141		1.00	2.24
7% CO_2_		45	60		39.5	52.7		53	135		1.00	2.55
		45	58		39.5	50.9		45	90		1.00	2.00
Mean		43.3	52.0									
Grand mean ([kPa])				22.7 (3.03)	39.5 (5.27)	51.5 (6.87)				0.67	1.00	1.71

*The arterial PaCO_2_ and CBF values from three human studies (Kety and Schmidt, 1946, 1948; Ramsay et al., 1993) are shown. The PaCO_2_ values for hyperventilation (H) control situation with normocapnia (C) and an experimental period (E) with special gas mixture enriched with 5–7% CO_2_ are shown in absolute values and relative (FH/C, FC/C, and FE/C) values normalized to standard average of F=39.5 mm Hg, taken from Ramsay et al. (1993). The CBF values for each situation (H, C, E) are shown in mL/100 g/min and as fraction of normocapnic flow (H/C, C/C, E/C)*.

(1) $$f_{C}^{\text{rhesus}}=0.42+\frac{\left(2.31-0.42\right)}{\left[1+B\;e^{k_{r}\log_{10}\text{PaC}{\text{O}}_{2}}\right]}$$

where *k_r_* = − 5.251 and *B* = 10,570. By changing the logarithm from log_10_ to the natural logarithm using log_10_(*x*) = ln(*x*)/ln(10) equation (1) becomes

(2) $$f_{C}^{\text{rhesus}}=0.42+\frac{\left(2.31-0.42\right)}{\left[1+B\;e^{\frac{k_{r}}{\text{In(10)}}{\text{*In(PaCO}}_{\text{2}}\text{)}}\right]}$$

This simplifies the equation to

(3) $$f_{C}^{\text{rhesus}}=0.42+\frac{\left(2.31-0.42\right)}{\left[1+B{\text{PaCO}}_{\text{2}}^{k_{a}}\right]}$$

where *k_a_* = *k*_r_/ln(10) = − 2.2805 and *B* = 10,570. This equation describes the CO_2_-induced relative CBF changes compared to the average baseline CBF and therefore serves as a basis for correction. The values 0.42 and 2.31 are the minimum and maximum relative change the blood flow is predicted to deviate from a normalized CBF of 1.

To fit this function to human data, we determined the values of *B* and *k_a_* and the maximum correction for hypercapnia *H*_max_, and the minimum correction for hypocapnia *H*_min_. Because the hypocapnic values of CBF have less variation, *H*_min_ represents an average relative decrease to 0.67 of the normocapnic flow. For hypercapnic values with greater variation of the CBF response, the theoretical maximum was chosen to be the maximally observed response of *H*_max_ = 2.55, i.e., 2.55 times the normocapnic flow. Given that the function must pass through a normocapnic correction factor of unity (at the point 39.5,1), *B* was isolated as a function of *k_a_*,

(4) $$B=\frac{H_{\text{max}}-1}{1-H_{\text{min}}}\;39.5^{-k_{a}}$$

**Figure 1:**
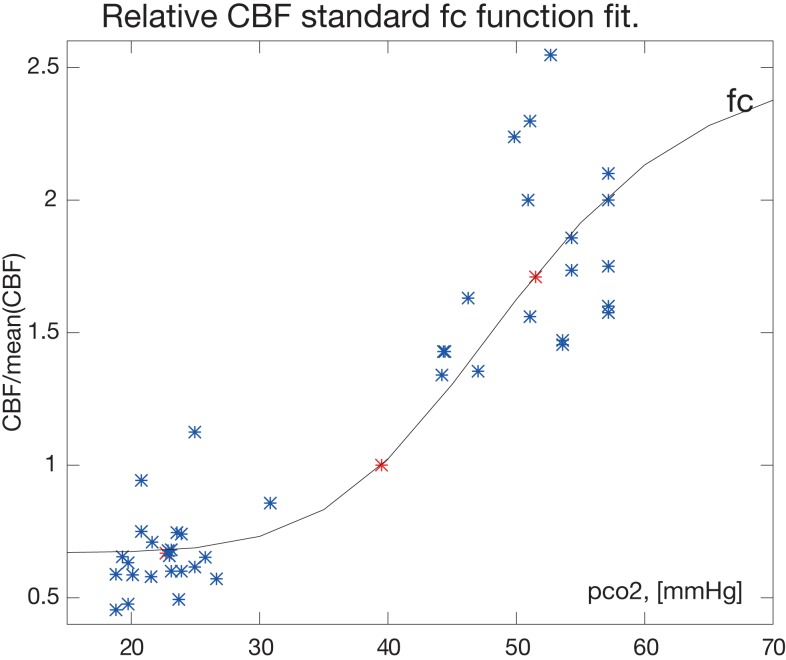
**The fitted function for the correction factor *f_c_***. The factor $f_{C}=0.67+\left(2.55-0.67\right)∕\left[1+2.34\times 10^{11}{\text{PaCO}}_{\text{2}}^{-\text{6}\text{.7}}\right]$ and the fractional CBF values H/C, C/C, and C/E are shown in relation to the relative PaCO_2_ (pco2) measure in [mmHg]. The normocapnic points constrain the fit at relative CBF value C/C = 1 by definition. Single measurements from Table 1 are blue, mean values are red (*).

This means that the correction factor *f_C_* is dependent only on the parameter constants *H*_min_, *H*_max_, and the remaining constant *k_a_*

(5) $$f_{C}=H_{\min}+\frac{\left(H_{\max}-H_{\min}\right)}{\left[1+B{\text{ PaCO}}_{\text{2}}^{k_{a}}\right]}$$

from which we estimate the remaining parameter *k_a_* by regression such that *f_C_* is consistent with the average values for the hypo- and hypercapnic response. The fitted function is shown in Figure 1.

The fitted version of equation (5) with fixed human parameters *H*_min_, *H*_max_, *B*, and *k_a_* is the standard curve used for the subsequent correction of new data. The standard curve assumes standard baseline CO_2_ set-point (*F*) for normocapnia to be at *F* = 39.5 mmHg.

#### Application of CO_2_ correction

The application of the standard correction factor to an actual dataset consist of tree main parts:

I) Find the baseline CO_2_ set-point $\left(\text{PaC}{\text{O’}}_{\text{2}}\right)$for the study, and define the scaling factor $R=F∕\text{PaC}{\text{O’}}_{\text{2}}$ relative to the standard *F* = 39.5 mmHg.
II) Scale the measured CO_2_ value ${\text{PaCO}}_{2}^{\text{measured}}$ by *R* and insert in equation (8) to find *f_C_*.
III) Divide the measured flow (CBF_measured_) by the *f_C_* to get the corrected flow CBF_corrected_.

For the application of the correction factor to an actual dataset, we need to know the baseline set-point ${(\text{PaCO’}}_{\text{2}})$ of average normal PaCO_2_ for that dataset. Note that this is possibly different in different subgroups, but was not statistically different in the groups we studied. Therefore we used a common mean $\text{PaC}{\text{O’}}_{\text{2}}$ for both AD and HC groups. This baseline set-point $\text{PaC}{\text{O’}}_{\text{2}}$ is used relative to the standard CO_2_ value *F* = 39.5 mmHg used in the regression to scale new CO_2_ values $\left({\text{PaCO}}_{\text{2}}^{\text{measured}}\right)$ relative to the standard CO_2_ range.

Having chosen the mean reference value $\text{PaC}{\text{O’}}_{\text{2}}$ whereby *R* becomes $R=F∕\text{PaC}{\text{O’}}_{2},$ the correction factor *f_C_* for a measured flow (CBF_measured_) observed at ${\text{PaCO}}_{2}^{\text{measured}}$ can be found as a modified version of equation (5) with normocapnia set at the $\text{PaC}{\text{O’}}_{\text{2}}$ value of the dataset,

(6) $$f_{C}=H_{\min}+\frac{\left(H_{\max}-H_{\min}\right)}{\left[1+B{(R{\text{ PaCO}}_{\text{2}}^{\text{measured}}\text{)}}^{k_{a}}\right]}$$

such that when ${\text{PaCO}}_{2}^{\text{measured}}=\text{PaC}{{\text{O}}^{\prime }}_{2},$ *f_C_* = 1, no correction occurs.

The actual corrected CBF value is now readily found by dividing the measured flow by the estimated *f_C_*.

(7) $$\text{CB}{\text{F}}_{\text{corrected}}=\frac{\text{CB}{\text{F}}_{\text{measured}}}{f_{C}}$$

For a given normocapnic value of CBF (CBF_norm_), the inverse predicted values for hypercapnia and hypocapnia CBF_predict_ can be found by the relationship,

(8) $$\text{CB}{\text{F}}_{\text{predict}}=f_{C}\;\text{CB}{\text{F}}_{\text{norm}}$$

### Positron emission tomography

During a series of PET investigations in which values of PaCO_2_ were not deliberately varied, we measured the arterial PaCO_2_ tension in samples from a group of healthy age-matched healthy volunteers (HC, n = 8) and a group of patients with Alzheimer’s disease (AD, n = 5). The tracers used to determine rates of CBF and CMRO_2_, respectively, were [^15^O]water and [^15^O]O_2._

#### Subjects

Five patients with AD (3 women, 2 men) with an average age of 64 (SD 7) years and moderately reduced Mini-mental State Examination (MMSE) scores of 22–25 volunteered to complete the tomography. The patients were recruited by the local Dementia Clinic and screened by an experienced neurologist for the presence of probable Dementia of Alzheimer’s Type (DAT). Eight healthy age-matched HC volunteers with MMSE and CAMCOG scores in the range of 28–30 with a mean age of 67 (SD = 6) years served as controls, recruited by public advertisement and screened with clinical, neurological, and neuropsychological testing including MMSE adapted to Danish (Lolk et al., 2000) to exclude cognitive impairment. We obtained written informed consent from all subjects to the protocols approved by the Regional Science Ethics Committee in accordance with the Declarations of Helsinki.

#### CBF and CMRO_2_

All subjects had one or two [^15^O]water and one or two [^15^O]O_2_ emission recordings in the 3D mode of the ECAT High Resolution Research Tomograph (HRRT, CTI/Siemens, Knoxville, TN, USA) in a quiet room with the subjects resting in a supine position with eyes open. The images were reconstructed with 3D-OP-OSEM point spread function reconstruction (Varrone et al., 2009) using 10 iterations and 16 subsets with FWHM at approximately 1.5 mm. The reconstructed images were corrected for random and scatter events, detector efficiency variations, and dead time. Tissue attenuation scans were performed using a rotating [^68^Ge] source. Dynamic emission recordings lasting 3 min (21 frames) were initiated upon bolus intravenous injection of [^15^O]water (500 MBq) or inhalation of [^15^O]O_2_ (1000 MBq). Catheters (Artflon and Venflon, Becton Dickinson, Swindon, UK) were inserted in the right radial artery and left cubital vein and arterial blood radioactivity was measured every half second for the duration of the PET scan by an automated blood sampling system (Allogg AB, Mariefred, Sweden), cross-calibrated with the tomograph, and then corrected for external delay and dispersion.

We quantified the CBF as the water clearance from the blood $K_{1}^{{\text{H}}_{2}\text{O}}$ in units of mL/100 g/min with the linearized two-compartment model (Blomqvist, 1984) modification of Ohta et al. (1996) and the Lawson-Hanson non-negative least squares solution to general least squares functions (Lawson and Hanson, 1974).

We quantified the CMRO_2_ in units of μmol/100 g/min from the oxygen clearance from the blood $K_{1}^{{\text{O}}_{2}}$ by multiplying individual hemoglobin concentrations with the oxygen clearance obtained with the same linearized two-compartment model (Blomqvist, 1984) modification of Ohta et al. (1992) and the Lawson-Hanson non-negative least squares solution to general least squares functions (Lawson and Hanson, 1974). Arterial CO_2_ tensions were measured in manually drawn blood samples obtained in relation to the PET acquisitions with the ABL825flex blood analyzer.

Individual images for each group were averaged after spatial normalization, to produce average CBF and average CMRO_2_ values. To enable direct comparison with the average CBF values, we scaled the average CMRO_2_ values back to the $K_{1}^{{\text{O}}_{2}}$ value in units of mL/100 g/min, using the group mean hemoglobin concentration Hb_mean_ for each subject.

(9) $$K_{1}^{{\text{O}}_{2}}={\text{CMR}}_{{\text{O}}_{2}}/{\text{Hb}}_{\text{mean}}\text{ and Δ}K_{1}=K_{1}^{{\text{H}}_{2}\text{O}}-K_{1}^{{\text{O}}_{2}}$$

Statistical difference between AD and HC group means were calculated for cortical values using a two-sample *t*-test of the null hypothesis of equal means. The analysis of variance between groups used the two-sample *F*-test of the null hypothesis of equal variances.

### Tissue oxygen gradient

In order to estimate the oxygen gradient in the tissue, we combined measures of CBF and CMRO_2_ to calculate the capillary oxygen tension and subsequently the CMRO_2_ to estimate the mitochondrial oxygen tension ${\text{P}}_{{\text{O}}_{\text{2}}}^{\text{mit}}$ decrease as a function of distance from the capillaries.

The derivation of the mitochondrial oxygen gradient in the tissue (Gjedde et al., 2002) proceeded as follows: The decline of oxygen tension from the capillaries to mitochondria determines the transport of oxygen and hence defines the rate of oxygen consumption (Gjedde et al., 2005),

(10) $${\text{CMR}}_{{\text{O}}_{2}}=L(P_{{\text{O}}_{2}}^{\text{cap}}-P_{{\text{O}}_{2}}^{\text{mit}})$$

where *L* is Krogh’s diffusion coefficient which depends on the distance from the capillaries, and $P_{{\text{O}}_{\text{2}}}^{\text{cap}}$ and $P_{{\text{O}}_{\text{2}}}^{\text{mit}}$ are the oxygen tensions in the capillaries and mitochondria, respectively. From this relationship, we defined the tissue gradient as a function of the diffusion capacity (Gjedde et al., 2010),

(11) $$P_{{\text{O}}_{2}}^{\text{mit}}=P_{{\text{O}}_{2}}^{\text{cap}}-\frac{1}{L}{\text{CMR}}_{{\text{O}}_{2}}$$

Given the average capillary oxygen tension $P_{{\text{O}}_{\text{2}}}^{\text{cap}}$ and the CMRO_2_ the mitochondrial oxygen tension $P_{{\text{O}}_{\text{2}}}^{\text{mit}}$ was plotted as a function of the diffusion capacity as shown in Figure 5.

$P_{{\text{O}}_{\text{2}}}^{\text{cap}}$ is the average capillary oxygen tension that depends on the hemoglobin saturation and satisfies the Hill equation. $P_{{\text{O}}_{\text{2}}}^{\text{cap}}$ can be expressed in terms of the half saturation constant of hemoglobin, $P_{\text{50}}^{\text{cap}},$ the Hill coefficient for capillaries *h*, and the extraction fraction of oxygen $E_{{\text{O}}_{\text{2}}}.$ For a complete derivation please see Chap. 22, pp. 523–549, in Gjedde (2005) and Vafaee and Gjedde (2000, 2004), Gjedde et al. (2005, 2010). This relation yields a formula that expresses the average capillary oxygen tension as a function of the oxygen extraction fraction and the hemoglobin oxygen dissociation curve, assuming 100% oxygen saturation of arterial hemoglobin,

(12) $$P_{{\text{O}}_{2}}^{\text{cap}}=P_{50}^{\text{cap}}\sqrt[{h}]{\frac{2}{E_{{\text{O}}_{2}}}-1}$$

which means that the capillary oxygen tension $P_{{\text{O}}_{\text{2}}}^{\text{cap}}$ can be calculated from the extraction fraction $E_{O}={\text{CMRO}}^{\text{2}}/(C_{{\text{O}}_{2}}^{\text{art}}\text{ CBF})$ (Gjedde et al., 2005) and by applying standard human values for $P_{\text{50}}^{\text{cap}}=27\;\text{mmHg}$ and *h* = 2.7. This formulation of $P_{{\text{O}}_{\text{2}}}^{\text{cap}}$ was used in equation (11) to express the tissue oxygen tension directly from the CBF and CMRO_2_

(13) $$P_{{\text{O}}_{2}}^{\text{mit}}=P_{50}^{\text{cap}}\sqrt[{h}]{\left[\frac{2C_{{\text{O}}_{2}}^{\text{art}}\text{CBF}}{{\text{CMR}}_{{\text{O}}_{2}}}-1\right]}-\left[\frac{1}{L}\right]{\text{CMR}}_{{\text{O}}_{2}}$$

where $C_{{\text{O}}_{\text{2}}}^{\text{art}}$ is the arterial concentration of oxygen, in this case assumed equal to the hemoglobin concentration for fully saturated arterial blood.

## Results

The results consist of three different parts:

1) The result of fitting the historic data and the parameters for the human correction factor. Application to subgroup data previously published.
2) The results of applying the standard correction method to a new dataset of AD and HC subjects.
3) Evaluation of oxygen gradients in order to explain the CBF influence on tissue oxygenation in AD.

### Correction for reactivity to CO_2_

To derive the equation that corrects for the PaCO_2_ variability in both groups, we adopted arterial PaCO_2_ and CBF values from three human studies reported in the literature (Kety and Schmidt, 1946, 1948; Ramsay et al., 1993), as listed in Table 1. These PaCO_2_ values refer to the hyperventilation (H), normocapnic (C), and experimental (E) conditions, where specific gas mixtures enriched with 5–7% CO_2_ yielded both with absolute values in units of mmHg and values relative to a standard average of 39.5 mmHg, adopted from Ramsay et al. (1993). The CBF values for each situation (H, C, E) are given in units of mL/100 g/min and as fractions of normocapnic flow (H/C, C/C, and E/C).

From the PaCO_2_ relative to the mean of 39.5 and fractional CBF values (i.e., *f_C_*), we fitted equation (5) to the H/C, C/C, and E/C response ratios reported in the range of 0.67- to 2.55-fold the average normal CBF (Figure 1). The parameters of the fitted standard function function 5 were *H*_min_ = 0.67, *H*_max′_ = 2.55, *B* = 2.34 × 10^11^, and *k_a_* = − 6.7.

In order to illustrate how this parameterized correction can be applied to individual data-sets with very different CBF group average and different mean CO_2_ level, we fitted the historic gray and white matter data separately using the steps (I), (II), and (III) previously described. The subgroup set-points used are listed in Table 2.

**Table 2:** **Normocapnic CBF measures (historic)**.

	$\text{PaC}{{\text{O}}^{\prime }}_{2}$ mmHg	CBF_norm_ mL/hg/min
Grey matter	44	60
White matter	40	19

**Figure 2:**
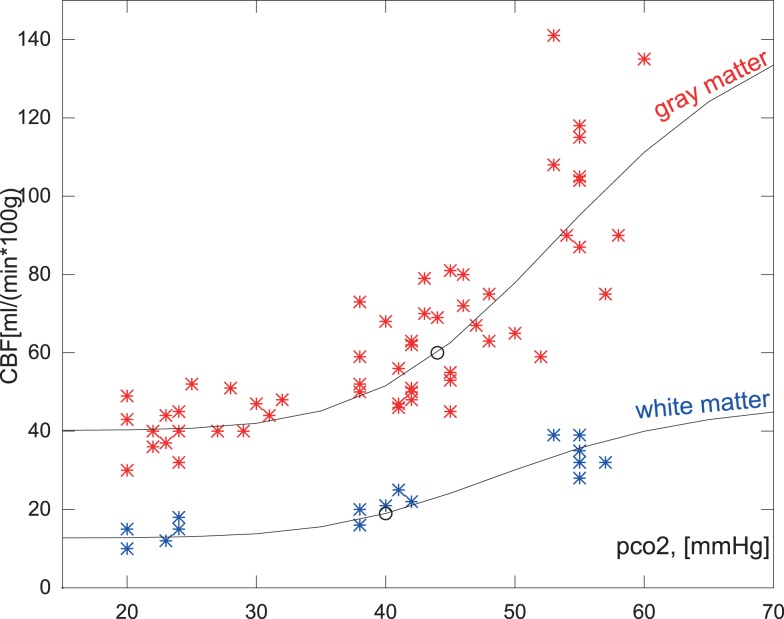
**Measured hypocapnic, normocapnic, and hypercapnic quantitative CBF values in relation to the absolute measured PaCO_2_ (pco2) in mm Hg**. The red values are cortical values taken from Ramsay et al. (1993) or measurements from Kety and Schmidt (1946, 1948), based on the nitrous oxide method. The blue values are white matter flow taken from Ramsay et al. (1993). The points are super-positioned with curves of the expected CBF values when using the correction function relative to a mean normocapnic flow.

As shown in Figure 2 the predicted the CBF in response to PaCO_2_ fits each of the subgroups well even though the data magnitudes involved are very different. A similar approach was used for the average values of regional values reported in Ito et al. (2008), as shown in Figure 6.

### CBF variability in AD and healthy aging

Subjects had spontaneous PaCO_2_ values as high as 45.2 mmHg and as low as 27.1 mmHg. Only data within the normocapnic range of 35–45 mm Hg was used to determine the normocapnic set-points. The mean $\left({\text{PaCO}}_{\text{2}}^{\text{measured}}\right)$ was 39.6 (SD 2.4) mmHg in the healthy subjects, and 38.3 (SD 0.9) mmHg in the AD patients. These group-wise normocapnic means were used to determine the CO_2_ response curves used for correction for CO_2_ by the steps (I), (II), and (III) described in the Methods.

**Figure 3:**
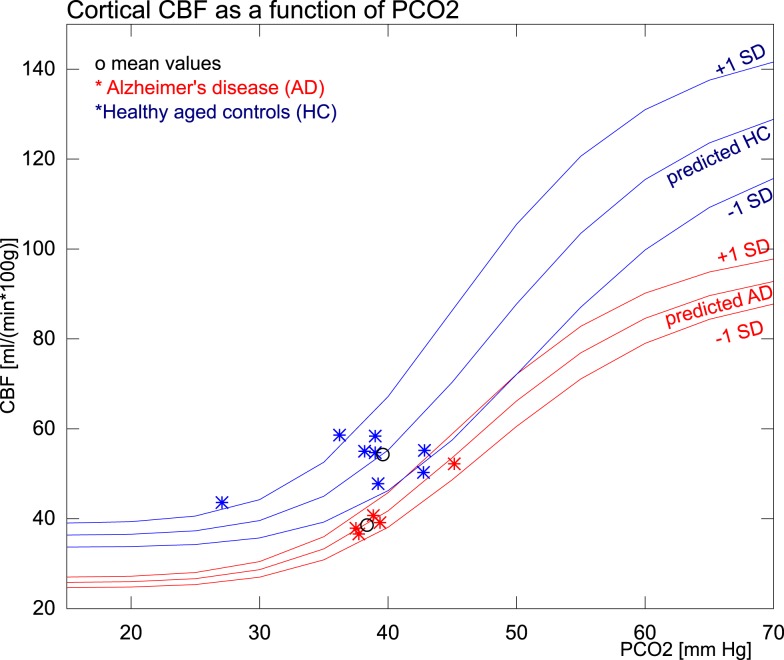
**The figure shows the measured and predicted CBF values for AD and healthy subjects in relation to PaCO_2_ measures**. The group means and SD used for the predictions are from within the normocapnic range of 35–45 mm Hg. The single SD estimates are based on $\left(\text{mea}{\text{n}}_{\text{pC}{\text{O}}_{\text{2}}}+1\text{S}{\text{D}}_{\text{pC}{\text{O}}_{\text{2}}},\text{mea}{\text{n}}_{cbf}+1{\text{SD}}_{{\text{pCO}}_{\text{2}}}\right)$ and $\left(\text{mea}{\text{n}}_{\text{pC}{\text{O}}_{\text{2}}}+1\text{S}{\text{D}}_{\text{pC}{\text{O}}_{\text{2}}},\text{mea}{\text{n}}_{cbf}-1\text{S}{\text{D}}_{\text{pC}{\text{O}}_{\text{2}}}\right)$.

CBF values for the AD and HC groups are reported in Table 3. Mean CBF was significantly lower in the AD group compared to the HC group, both with (*p* < 0.0005) and without (*p* < 0.005) correction for CO_2_. The variation, however, was only significantly reduced in the AD group when data had been corrected for CO_2_ differences. The PaCO_2_ corrections indicated that substantial variability of CBF stemmed from the PaCO_2_ effect in both groups. Figure 3 shows the corresponding values of PaCO_2_ and CBF for the two groups. The healthy controls had considerable additional variation of CBF, which was not related to differences of PaCO_2_. In the AD patients, however, the average CBF was substantially lower and the entire variation was commensurate with the response predicted by PaCO_2_.

**Table 3:** **CBF before and after correction for CO_2_**.

	PaCO_2_ mmHg	CBF mL/hg/min	CBF_corrected_ mL/hg/min	CMRO_2_ μmol/hg/min
AD	45.2	52	37	185
	39.4	39	37	149
	37.5	38	39	153
	38.9	41	40	151
	37.7	37	38	160
Mean	39.7 (3.1)	41(6.3)**	38(1.2**)***	159(15)
Mean (35–45 mmHg)	38.4 (0.9)	38.6(1.7)***	39(1.2*)*	
HC	39.000	55	56	187
	39.225	48	49	190
	38.175	55	59	148
	27.075	44	62	210
	39.000	58	60	200
	36.225	59	68	209
	42.750	50	43	158
	42.825	55	47	154
Mean	38.0(5.0)	53(5.3)	56(8.4)	182(25)
Mean (35–45 mmHg)	39.6(2.4)	54(4.0)	55(8.5)	

Group comparisons were done for difference between AD and HC *t*-test of means and a *f*-test was done for difference in variance. The AD had significant decreased mean and variance after correction for the CO_2_ effect (***p < 0.0005, **p < 0.005, *p < 0.05).

### CBF variability and tissue oxygenation

**Figure 4:**
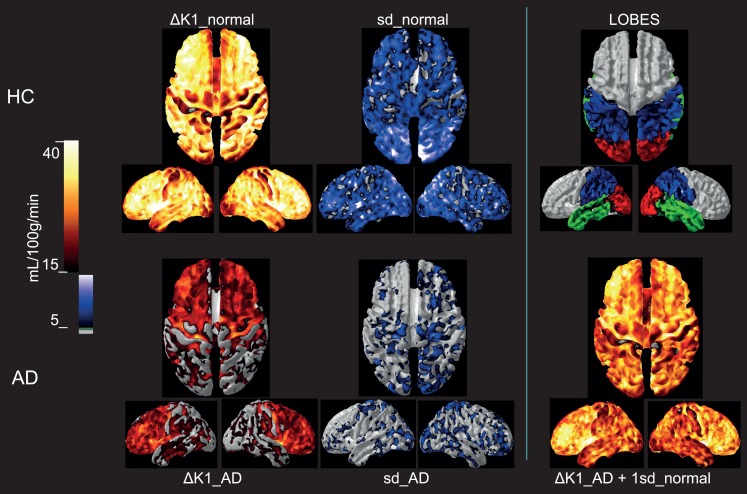
***Upper row:* Group mean Δ*K*_1_ and 1 SD images for the healthy control subjects as well as a lobe overview, *Lower row:* Group mean Δ*K*_1_ and 1 SD images for the Alzheimer’s patients**. For comparison is added in the third column a calculated image of adding 1 SD of the healthy aged controls to the Δ*K*_1_ magnitude of the AD. Group mean Δ*K*_1_ images are colored using hot-metal color scale (values below 15 mL/100 g/min in gray). 1 SD images are shown in black, blue, and white color scale (values below 5 mL/100 g/min in gray). The lobe overview indicates the position of temporal lobes in green, occipital lobes in red and parietal lobes in blue. The standard deviations for AD patients were much lower than for the controls, and the group difference in Δ*K*_1_ possibly could be explained by the loss of variability in the patients. All images are mapped onto standard brain gray-white matter surfaces using FACE software from Aalborg University (Eskildsen and Østergaard, 2006).

To evaluate the extent to which the loss of additional variability of CBF in the AD patients is explained by uncoupling of CBF from oxygen metabolism, we determined the difference between the blood-brain clearances (*K*_1_) of water and oxygen (Δ*K*_1_), i.e., the component of CBF not devoted to the clearance of oxygen. As shown in Figure 4, for healthy controls, the association (non-primary) cortices of the frontal, occipital, parietal, and temporal lobes have the highest Δ*K*_1_ values, while *K*_1_ values in primary sensorimotor cortices were much closer to each other, also indicative of greater oxygen extraction. However, for the standard deviation among the HC subjects, rendered as a color-coded map shown in Figure 4, the higher variability of Δ*K*_1_ is evident in occipital, parietal, and temporal lobes compared to the frontal lobe.

**Figure 5:**
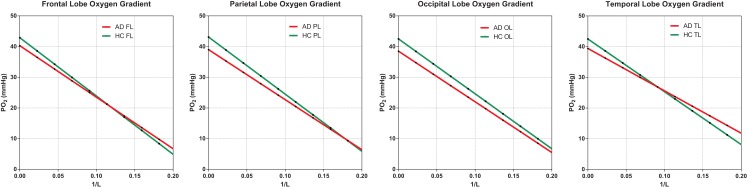
**Oxygen gradient plotted through the tissue**. AD gradients are not significantly below HC gradients, indicating no shortage of oxygen due to insufficient oxygen delivery. The assumption here is normal or high capillary density, translated into diffusion capacity (L), as generally observed in the literature. Capillary density would have to be significantly reduced to suggest hypoxic conditions in AD. The capillary oxygen tension is the oxygen tension for 1/L ; 0.0.

Average capillary oxygen tensions, calculated from equation (12), declined significantly in the AD patients compared to the age-matched HC subjects, as shown in Figure 5. In both groups, the decline of oxygen in the tissue depended on the degree of non-linear coupling of flow to oxygen consumption, and the resulting gradients of oxygen tension determined from equation (13) did not differ significantly between the groups and thus did not indicate deficient oxygen delivery in AD.

## Discussion

### General CO_2_ correction method

The method presented here is a practical approach to the correction of CBF data for changes of PaCO_2_ that relates individual measures to the normocapnic mean for an individual or a group. The correction factor is derived from historical data obtained from a broad spectrum of studies. The correction is independent of the absolute flow rates and thus applicable to both grey and white matter regions of the brain.

The range of PaCO_2_ values is established by intra-individual responses to the tomography such as anxiety-induced hyperventilation, and inter-individual and group variations of PaCO_2_, and the present approach accounts for all of these changes. In studies analyzed by general linear model (GLM) statistics, PaCO_2_ variation can be factored out as a covariate, but the procedure considers only the influence on the statistics, not the magnitude of the corrected flow. The GLM model is not appropriate for modeling non-linear relationships. Another approach to PaCO_2_ correction is a simple division of the CBF measure with the PaCO_2_ values (Ashwal et al., 1991), but the procedure yields a linear relationship and therefore does not account for the differences of the CBF/mmHg ratio in hypocapnia compared to hypercapnia. The result is consistent with hypocapnic reactivity but underestimates the hypercapnic response. Claassen et al. (2007) also applied a logistic model somewhat similar to the one introduced here. However it was applied to CBFV data and not CBF data at steady state, but they showed a good agreement with the logistic shape even at very high time resolution.

Although the methods of measuring and correcting for the hemodynamic response to changes of the arterial CO_2_ tension vary somewhat, the results of separate studies in man agree to a considerable extent when interpreted relative to normocapnic values, as demonstrated in Figure 1. In this figure, data extracted from three different human studies are represented. Data from references Kety and Schmidt (1946) and Kety and Schmidt (1948) include measures of healthy young men in whom control values of CBF were determined with the nitrous oxide method of Kety and Schmidt at normal ambient conditions and normal breathing. The effects of passive and active hyperventilation provided hypocapnic values for the same subjects. For hypercapnia, the subjects were exposed to an atmosphere of 21% oxygen with 5 or 7% added CO_2_. Intra-subject CBF measures with varying CO_2_ tensions were also reported by Ramsay et al. (1993). Because they also used PET to determine CBF, we adopted their mean normocapnic PaCO_2_ of 39.5 mmHg as the standard for which the correction factor is unity in the present PET study.

We modeled the data by the function originally introduced by Reivich (1964), which included theoretical values for maximum vasodilatation and -constriction. Although these values are uncertain in man, the functional relationship obtained with the parameter values proposed here fits the data well.

In primates, Reivich (1964) found the maximum response to be 42% of the average normal CBF of 49.3 mL/100 g/min for vasoconstriction and 231% of the average normal CBF for vasodilatation, corresponding to CO_2_ tensions of 15 mmHg (2 kPa) and 150 mm Hg (20 kPa), respectively.

In contrast, in man, measured extremes of PaCO_2_ are closer to 20 and 60 mmHg (Kety and Schmidt, 1946, 1948; Ramsay et al., 1993), corresponding to a measured range of response of 44–255% of the average normal CBF value. As the response in rhesus monkeys appears to be less than in humans, a CO_2_ tension of 60 mmHg yields a 146% increase of CBF in the monkeys but a 218% increase in humans, where the same increase is reached at 48 mmHg. In primates, the sensitivity to CO_2_ fits a logistic exponential relationship between carbon dioxide tensions and blood flow, and we chose to modify this relationship with different parameters descriptive of the human situation as the overall functional shape for humans.

#### Regional variation

**Figure 6:**
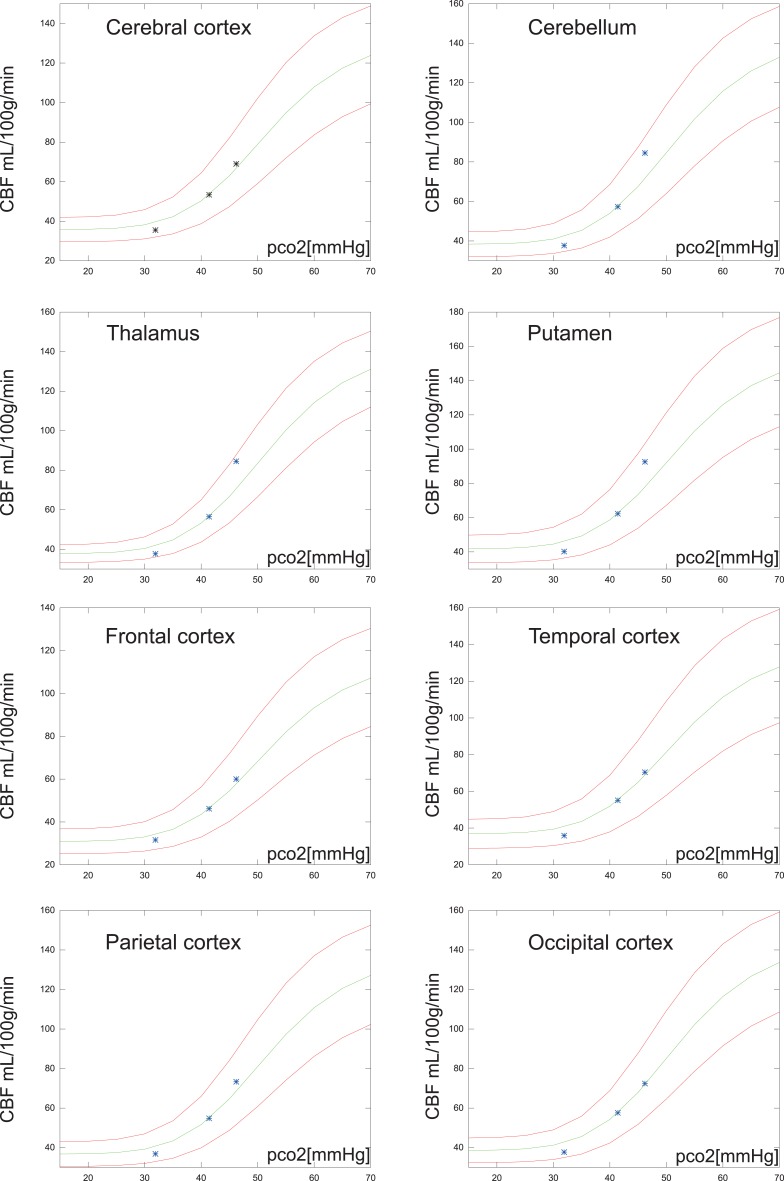
**Regional average CBF values (N = 20) of the hypocapnia, normocapnia, and hypercapnia conditions**. The green line is the estimated CBF based on application of the correction factor to the normocapnic values of PaCO_2_ and CBF. The red lines are estimates of CBF response, 95% confidence, i.e., 1.96 SD from normocapnic values of PaCO_2_ and CBF.

The relationship among baseline CBF values and the regional vascular PaCO_2_ responses recently was revisited by Ito et al. (2008). Instead of individual numbers, the authors reported regional average CBF values for 20 subjects at hypo-, normo-, and hypercapnia. The correction proposed here is in good agreement with cortex values, but cerebellum, thalamus, and putamen had slightly higher vascular responses than those estimated from the present correction factor as shown in Figure 6.

### Correction for AD patients and control subjects

In the HC group estimates of the vasomotor response to CO_2_, the normal flow variation in the 35- to 45-mmHg PaCO_2_ range is variable and not explained by the predicted response to CO_2_. This discrepancy reflects the number of factors suspected of influencing CBF. In the AD group, however, we found very little variation in the normocapnic range and the variability of CBF was closely coupled to the predicted response. The markedly decreased variability in AD is consistent with a blunting of the general neurovascular control unit variability, with largely conserved CO_2_ regulation.

In a recent MRI study of familiar frontotemporal dementia (FTD-3) prior to symptom onset, the authors found a significant decrease of CBF in temporal, occipital, and parietal regions of familial carriers of the CHMP2B mutation, compared to family members not carrying the mutation. However, the reduction was observed only with spin echo sequences sensitive to deoxyhemoglobin in the capillary bed, suggesting that capillaries rather than arterioles are the mediators of the decrease, as befits a functional decline (Lunau et al., 2012). The carriers of the mutation had a marked loss of CBF variability in the same regions (temporal, occipital, and parietal regions) found to have declines of the Δ*K*_1_ estimates in the present study. Although the dementia of that study is different from the AD studied here, the observation is consistent with the claim that these regions are particularly important and vulnerable to the development of early signs of dementia.

#### Is Δ*K*_1_ important to glucose delivery?

As averaged for each group, the variability of the Δ*K*_1_ measure is related to the BOLD signal obtained with functional MRI, although it has lower time resolution. For the Δ*K*_1_ shown in Figure 4, much of the variation appears to be lost in the AD patients, as the Δ*K*_1_ magnitude is decreased overall but especially in the occipital, parietal, and temporal lobes. To illustrate how the loss of variability of the HC group coincides with the loss of Δ*K*_1_ for the AD group, we show the result of adding 1 SD of the normal variation to the low measure of the AD patients. The calculated AD image strikingly fits the distribution of the Δ*K*_1_ estimates of the age-matched HC controls. Although this has no direct physiological meaning it illustrates that the difference between AD and HC subjects may be correlated to a loss of CBF variability.

We speculate that this loss may by some means be related to the loss of ability or necessity of the vasculature to adjust the CBF to functional demands, especially in the posterior and temporal regions of the brain.

An important question is whether the lack of variability expressed as the Δ*K*_1_ estimate is consistent with an adequate supply of oxygen, or adequate transport of substrate such as glucose.

As for glucose, recent reports (Vaishnavi et al., 2010; Vlassenko et al., 2010) suggest that aerobic glycolysis in healthy young adults is spatially correlated with the depositions of amyloid-β in AD patients. The authors suggest the existence of a link between high rates of aerobic glycolysis in young adults and the later development of AD pathology. In the present study, the values of Δ*K*_1_ may suggest that CBF may normally be uncoupled from metabolism in the areas with higher aerobic glycolysis indices (Vaishnavi et al., 2010; Vlassenko et al., 2010) in the age-matched HC group. In the AD group, however, the loss of the Δ*K*_1_ reserve clearly is more pronounced in the posterior and temporal parts of the brain, as previously described.

Amyloid beta (Aβ) deposition has been implicated in decreased glucose transporter-1 levels and hippocampal atrophy in brains of aged APP/PS1 mice (Hooijmans et al., 2007) at the capillary level without decreased capillary density. The mechanism could be an interplay between the upstream Aβ oligomers and neurotrophic factors such as proNGF/NGF (Schliebs and Arendt, 2011), as neurotrophic factors also have been implicated in the reduction of GLUT-1 carrier density in the endothelial cells (Farkas and Luiten, 2001). With the exception of putamen and cerebellum, hexose transporters regionally were found to be about half as numerous in post mortem AD brains as in control brains (Kalaria and Harik, 1989).

Another indicator that the decrease of Δ*K*_1_ is consistent with a loss of aerobic glycolytic capability, is the reported observations that the absolute number of mitochondria in endothelial cells is conserved in AD, although the density per unit volume decreased due to endothelial basal membrane swelling or mitochondrial shrinkage, both implying impaired blood-brain barrier (BBB) capacity (Mancardi et al., 1985; Stewart et al., 1992) for glucose transport but not for the free diffusion of O_2_. We note here that microvascular basal membrane pathology in AD with the accumulation of laminin and heparan sulfate proteoglycans (HSPGs) in some studies has been linked to the perivascular astrocytes (Farkas and Luiten, 2001) which are more glucose dependent that neurons (Bolaños et al., 2010).

#### Is Δ*K*_1_ important to oxygen delivery?

For oxygen, the absent decline of capillary density in AD (Richard et al., 2010) allowed us to compare predicted oxygen gradients in the groups of AD patients and age-matched HC subjects and to show that the steady state gradients were nearly identical (Figure 5), except for the slightly lower capillary oxygen tensions associated with the higher oxygen extraction fractions of the AD patients. The lower oxygen tensions reflect the non-linearity of the relation between blood flow and oxygen consumption (and hence also the BOLD signal and the inter-individual Δ*K*_1_) that generally follows the ratio of oxy- to deoxyhemoglobin and therefore also elevates the oxygen extraction fraction when oxygen consumption declines, or lowers the extraction when oxygen consumption increases relative to blood flow during functional activation. Recent findings are consistent with the claim that the resulting O_2_ overshoot during activation prevents a sustained drop of oxygenation at tissue locations that are remote from the capillaries (Vafaee et al., 1999; Vafaee and Gjedde, 2000; Gjedde et al., 2005; Devor et al., 2011). The equivalent gradients of oxygen in the AD and HC subjects indicate that oxygen delivery at steady state is adequate for the attenuated functional excursions of brains with Alzheimer’s disease.

### Limitations

The results could be influenced by changes in blood pressure and hemoglobin oxygenation (Rasmussen et al., 2007; van Beek et al., 2012). Also direct effects of CO_2_ on brain functional variability itself are possible (Xu et al., 2011).

## Conflict of Interest Statement

The authors declare that the research was conducted in the absence of any commercial or financial relationships that could be construed as a potential conflict of interest.

## Footnotes

^1^A matlab^®^ subroutine and a CBF image scaling script for the MINC format is freely distributed by email request to the corresponding author.
